# Switching Adaptive Control with Applications on Robot Manipulators

**DOI:** 10.3390/e24101360

**Published:** 2022-09-25

**Authors:** Shihao Wang, Shiqi Zheng, Yushu Deng, Zhouxiang Jiang, Bao Song, Xiaoqi Tang

**Affiliations:** 1 School of Automation, China University of Geosciences, Wuhan 430074, China; 2 Shaoyang Institute of Advanced Manufacturing Technology, Shaoyang 422000, China; 3 Institute of Electromechanical Engineering, Beijing Information Science & Technology University, 12 Xiaoying East Road, Qinghe, Haidian District, Beijing 100192, China; 4 School of Mechanical Science and Engineering, Huazhong University of Science and Technology, Wuhan 430074, China

**Keywords:** adaptive control, logic-based switching, finite-time control, sampled-data control

## Abstract

This paper concentrates on the study of logic-based switching adaptive control. Two different cases will be considered. In the first case, the finite time stabilization problem for a class of nonlinear system is studied. Based on the recently developed adding a barrier power integrator technique, a new logic-based switching adaptive control method is proposed. In contrast with the existing results, finite time stability can be achieved when the considered systems contain both fully unknown nonlinearties and unknown control direction. Moreover, the proposed controller has a very simple structure and no approximation methods, e.g., neural networks/fuzzy logic, are needed. In the second case, the sampled-data control for a class of nonlinear system is investigated. New sampled-data logic-based switching mechanism is proposed. Compared with previous works, the considered nonlinear system has an uncertain linear growth rate. The control parameters and the sampling time can be adjusted adaptively to render the exponential stability of the closed loop system. Applications in robot manipulators are conducted to verify the proposed results.

## 1. Introduction

Adaptive control method has drawn an increasing interest both from scientific and engineering point of view during the past decades [1,2]. The major advantage of adaptive control lies on its unique capabilities to deal with various kinds of uncertainties in complex systems, such as variations of inertia in servo motor and robot systems [3], component faults in unmanned aircraft and ships, and unknown nonlinearties in flexible distributed parameter systems [4,5].

Due to the theoretical and practical significance of adaptive control, different kinds of adaptive controllers have been proposed. For linear systems, model reference adaptive control (MRAC) has been widely studied. The main idea of MRAC is to match the closed-loop systems to a reference systems [2]. For nonlinear systems, adaptive backstepping method is the most commonly technique [6,7,8]. It is a systematic design method that can compute the controller in an iterative way. For example, by using fuzzy logic, an adaptive backstepping control method has been proposed for non-strict feedback stochastic nonlinear systems [6].

Recently, a class of logic-based switching adaptive control (LSAC) has drawn an increasing attention [6,7,8,9,10,11]. The idea of LSAC is to adaptively tune the controller parameters by using a supervisory function and logic-based switching rule. LSAC enjoys several distinguishing features and may overcome some limitations in traditional adaptive control. For instances, it can well handle the loss of stabilizability phenomenon. Exponential/finite time stability can be achieved for high order nonlinear systems with unknown control directions [9,12].

On this note, we will take a further study on the logic-based switching adaptive control. Specifically, we will consider two different cases.

In the first case, we will consider the finite time stabilization problem for a class of nonlinear system. Based on the recently developed adding a barrier power integrator technique, a new logic-based switching adaptive control method is proposed. In contrast with the existing results, e.g., refs. [9,12], finite/fixed time stability can be achieved when the considered systems contain both fully unknown nonlinearties and unknown control direction. Moreover, the proposed controller has a very simple structure and no approximation methods, e.g., neural networks/fuzzy logic, are needed.

In the second case, we will consider the sampled-data control for a class of nonlinear system. New sampled-data logic-based switching mechanism is proposed. Compared with previous works, e.g., refs. [12,13], the considered nonlinear system has an uncertain linear growth rate. The control parameters and the sampling time can be adjusted adaptively to render the exponential stability of the closed loop system.

The following parts will be organized as follows. Section 2 will give a detailed literature review. Finite time and sampled data control will be discussed in Section 3 and Section 4, respectively. Simulations are conducted in Section 5. Section 6 concludes the paper.

## 2. Related Works

### 2.1. Finite-Time Control

The purpose of the finite-time control is to drive the states to zero in finite time [14]. Compared with traditional asymptotic control, finite time control can obtain higher control accuracy and faster convergence speed. Hence, it has been widely used in many industrial fields, such as the control of robot manipulators and CNC machines.

Many excellent results have been obtained in recent years. A typical method is to add some power terms into the controller, which will render finite-time stability. For examples, in [15], the authors investigated the finite/fixed-time stability of discontinuous systems. In order to improve the control performance of servo motors, a nonsingular fast terminal sliding mode control method with double power terms was proposed in [16]. Kong et al. [17] presented a new finite-time control method for robots with actuator saturation and workspace constraints.

Unknown control directions are often encountered in real engineering world. It means that the sign of control coefficient is unknown. The control methods can be roughly classified into two classes: Nussbaum-gain methods and logic-based switching methods. The idea of Nussbaum-gain method is to introduce a sine/cosine-like functions that can maintain the stability of the closed-loop systems. For instance, Lv et al. [18] studied the consensus tracking problem for a class of nonlinear multi-agent systems with partially unknown control directions. New Nussbaum-gain methods were carefully designed to handle various unknown control directions. Liang et al. [19] proposed a fractional Nussbaum-gain technique to handle fractional-order systems with unknown control directions. Nevertheless, the Nussbaum-gain technique cannnot achieve finite-time stability. Only asymptotic stability can be obtained.

The concept of logic-based switching methods is to adopt a logic-based switching rule to tune the controller parameters online according to a well-defined supervisory function. In [12], a new adaptive control method was proposed to achieve finite-time stability despite unknown control directions. However, the above methods can only be used to deal with nonlinear system with parametric uncertainties. In real practical applications, the system may contain some very complex uncertainties that cannot be described exactly, that is nonparametric uncertainties. More specifically, the form and parameters ϑ of the unknown nonlinearities f(x,ϑ) in the system are both unknown. To the best of our knowledge, few studies have been taken for the finite/fixed-time control problem for nonlinear systems with nonparametric uncertainties.

### 2.2. Sampled-Data Control

Many researchers have conducted a lot of work about the sampled-data control problem. The methods can be divided into backstepping, feedback domain and LMI (Linear Matrix Inequalities) methods. In [20], the authors developed a backstepping sampled data control method for a flexible robotic manipulator whose internal dynamic is completely unknown. In [21], the authors proposed a feedback domain method for sampled-data nonlinear systems. They also extended the work to more general systems, such as interconnected system, stochastic systems and disturbed systems. Based on the input-delay method, LMI conditions were proposed to check the stability of sampled-data control systems.

Though the above works have made good contributions to the sampled data control, there are at least two issues that can be improved. First, the linear growth rate θ for $f_{i}\left({\bar{x}}_{i}\right)$ is assumed to be *known*. That is $|f_{i}\left({\bar{x}}_{i}\right)|\leq \theta \varphi \left({\bar{x}}_{i}\right)$ where $\varphi ({\bar{x}}_{i})$ is an unknown function. However, due to the existence of uncertainties, a natural question is whether we can solve the sampled data stabilization problem for systems with *unknown* θ. Second, as stated in [21], it is not easy to compute an appropriate upper bound for the sampling period even if θ is known. The computed upper bound is usually conservative. Moreover, when θ is unknown, there will be no tools available to compute the sampling period.

Motivated by the above thought, this paper will propose an adaptive sampled data control strategy for system (13) with unknown growth rate θ. A logic-based switching law will be proposed to tune the controller parameters and the sampling period online simultaneously. We will show that after a finite number of switchings, all the states will converge to zero exponentially and the sampling period will be determined automatically.

## 3. Finite Time Control for Uncertain Nonlinear System with Unknown Control Direction

Consider the following first-order nonlinear system. Note that our method can be extended to more general cases, such as normal or strict-feedback nonlinear systems.

(1) $$\begin{array}{cc} \dot{\chi } & =b(\chi )u+f(\chi ) \end{array}$$

where χ∈R is the state variable, f(χ),b(χ) are unknown continuously differentiable nonlinear functions such that f(0)≡0 and |b(χ)|>0. The sign of b(x) is also unknown and *u* is the control input. The control objective aims to make the state *x* converge to zero in finite time. It is noted that using Taylor expansion we can conclude that there exist a smooth unknown function ϕ(χ) such that |f(χ)|≤|χ|ϕ(χ).

The proposed control method will be developed in the following three subsections. In Section 3.1, the structure of the controller will be designed, which contains a switching adaptive parameter $\hat{\theta }\left(t\right)$. In Section 3.2, the detail switching rule for the adaptive parameter will be presented. Section 3.3 gives the stability analysis.

### 3.1. Controller Design

First, consider the following Lyapunov function

(2) $$V=\frac{1}{2}\ln\left(\frac{{\bar{\chi }}^{2}}{{\bar{\chi }}^{2}-\chi^{2}}\right)$$

where $\bar{\chi }>0$ is a positive constant, $|\chi \left(0\right)|<\bar{\chi }$.

Notably, *V* is a barrier Lyapunov function such that if $|\chi |<\bar{\chi }$, then V→+∞ as $\left|\chi \right|\to \bar{\chi }$. The positive constant $\bar{\chi }$ acts as a barrier for χ. The purpose of adopting the barrier Lyapunov function is to constrain the state χ in the interval $\left(-\bar{\chi },\bar{\chi }\right)$. Note that as long as *V* is bounded, $\chi \in (-\bar{\chi },\bar{\chi })$.

In the following design, we assume $|\chi |<\bar{\chi }$. This will be verified later in the stability analysis in Section 3.3.

Differentiating *V* with respect to time, using system dynamic (1), we have

(3) $$\begin{array}{cc} \dot{V}\leq & \frac{\chi (bu+f(\chi ))}{{\bar{\chi }}^{2}-\chi^{2}}\leq \frac{\chi bu+\chi^{2}\phi \left(\chi \right)}{{\bar{\chi }}^{2}-\chi^{2}}\\ \leq & \frac{\chi bu}{{\bar{\chi }}^{2}-\chi^{2}}+\frac{U\chi^{1+\alpha }F\left(\chi ,\bar{\chi }\right)}{{\left({\bar{\chi }}^{2}-\chi^{2}\right)}^{2+2\alpha }} \end{array}$$

where $\alpha \in (\frac{1}{2},1)$ is a ratio of odd integers, U>0 is a design parameter, ϕ(x) and $F\left(x,\chi \right)=\chi^{1-q}\phi \left(x\right){\left({\bar{\chi }}^{2}-\chi^{2}\right)}^{1+2\alpha }/U$ are unknown functions caused by unstructured uncertainties.

Then, we design the control effort *u* as

(4) $$u=\hat{\theta }\left(t\right)\left[-K\chi^{\alpha }-L\chi^{\beta }-\frac{U\chi^{\alpha }}{{\left({\bar{\chi }}^{2}-\chi^{2}\right)}^{1+2\alpha }}\right]$$

where K,L are design parameters, β∈(1,+∞). $\hat{\theta }$ is an adaptive parameter. It will vary according to a switching signal σ(t).

(5) $$\hat{\theta }\left(t\right)={\left(-1\right)}^{\sigma (t)}\upsilon \left(\sigma \left(t\right)\right)$$

where σ(t):[0,+∞)→N is a non-decreasing piecewise constant switching signal. υ(σ):N→R is an increasing function with respect to σ such that υ(0)>0 and υ(σ)→+∞ as σ→+∞. A typical example of θ(σ) is υ(0)=1,υ(1)=2,υ(2)=3,… The idea of tuning rule (5) for $\hat{\theta }$ is that by changing its sign repeatedly, one may expect to find a correct control direction. The detail switching rule for $\hat{\theta }$ will be given in Section 3.2. For now, the switching signal σ and the adaptive parameter $\hat{\theta }$ are regarded as constants. This will become clear in Section 3.3.

Substituting (4) into (3), we get

(6) $$\begin{array}{cc} \dot{V}\leq & -\frac{K^{\prime }\chi^{1+\alpha }}{{\bar{\chi }}^{2}-\chi^{2}}-\frac{L\chi^{1+\beta }}{{\bar{\chi }}^{2}-\chi^{2}}+\frac{\left(K^{\prime }-bK\hat{\theta }\right)x^{1+\alpha }}{{\bar{\chi }}^{2}-\chi^{2}}\\ \; & +\frac{U\left(F\left(\chi ,\bar{\chi }\right)-h\hat{\theta }\right)s^{1+\alpha }}{{\left({\bar{\chi }}^{2}-\chi^{2}\right)}^{2+2\alpha }} \end{array}$$

where $K^{\prime }>0$ is positive constant.

From the above inequality, we can see that if $|\chi |<\bar{\chi }$, then there exist unknown positive constants $\bar{F},\underline{b}$ such that $F\left(\chi ,\bar{\chi }\right)\leq \bar{F}$ and $\left|b\left(\chi \right)\right|\geq \underline{b}>0$. Thus, we can deal with the unstructured uncertainties similar to structured uncertainties. That is, Equation (6) can be put in the following form when $|\chi |<\bar{\chi }$.

(7) $$\begin{array}{cc} \dot{V}\leq & -\frac{K^{\prime }\chi^{1+\alpha }}{{\bar{\chi }}^{2}-\chi^{2}}-\frac{L\chi^{1+\beta }}{{\bar{\chi }}^{2}-\chi^{2}}+\frac{\left(K^{\prime }-bK\hat{\theta }\right)x^{1+\alpha }}{{\bar{\chi }}^{2}-\chi^{2}}\\ \; & +\frac{U\left(\bar{F}-b\hat{\theta }\right)s^{1+\alpha }}{{\left({\bar{\chi }}^{2}-\chi^{2}\right)}^{2+2\alpha }} \end{array}$$

with $\left|b\right|\geq \underline{b}>0$. We can see that the unknown function $F(\chi ,\bar{\chi })$ is replaced by an unknown parameter $\bar{F}$. Meanwhile, the unknown function b(χ) is larger than an unknown positive constant $\underline{b}$. This result is similar to the work in [9] for parametric uncertainties.

Moreover, from (5), we find that there exists a sufficiently large integer σ such that

$$\begin{array}{cc} \mathrm{sgn}(\hat{\theta }) & =\mathrm{sgn}(b(x)),\\ K^{\prime }-bK\hat{\theta } & \leq K^{\prime }-\underline{b}K\hat{\theta }<0,\\ \bar{F}-b\hat{\theta } & \leq \bar{F}-\underline{b}\hat{\theta }<0. \end{array}$$

Then, (7) becomes

(8) $$\begin{array}{cc} \dot{V}\leq -\frac{K^{\prime }\chi^{1+\alpha }}{{\bar{\chi }}^{2}-\chi^{2}}\leq & -aV^{\gamma } \end{array}$$

where a>0 is a positive constant. $\gamma =\frac{1+\alpha }{2}\in \left(\frac{1}{2},1\right)$.

According to the above analysis, we can see (6) and (7) hold when $\sigma ,\hat{\theta }$ are regarded as constants and $|\chi \left(t\right)|<\bar{\chi }$. Moreover, when σ is sufficiently large in (5) and $|\chi \left(t\right)|<\bar{\chi }$, we also have (8). These results will be used in the stability analysis.

### 3.2. Logic-Based Switching Rule

The logic-based switching mechanism is shown in Algorithm 1. The switching signal σ is guided by a supervisory function S(·) defined as:

(9) $$\begin{array}{cc} S(\cdot ) & ≜V(\cdot )-\zeta (t), \end{array}$$

(10) $$\begin{array}{cc} \dot{\zeta }\left(t\right) & =-a\zeta^{\gamma } \end{array}$$

where ζ is an auxiliary variable, *a* is defined in (8).

**Figure 1:**
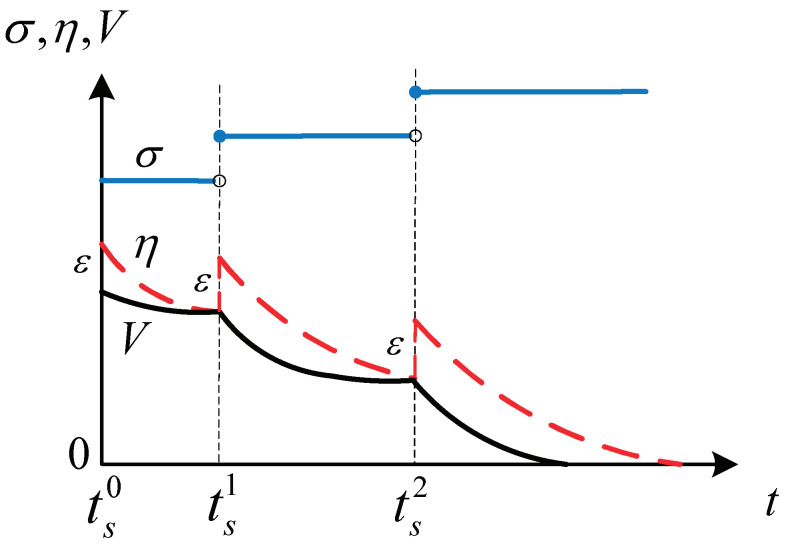
Variations of σ,η,V.

The idea of the algorithm is as follows. As shown in Figure 1, at each time instant, we verify whether or not the supervisory function S(·) is larger than zero. If not, then the switching signal σ remains constant; otherwise, the switching signal σ is increased by one and the adaptive parameter $\hat{\theta }\left(t\right)$ is updated by (5). Meanwhile, we reset ζ to make sure it is larger than *V*. This will avoid the situation where the parameters are updated repeatedly.

In more detail, at each switching time $t_{s}^{m}\left(m=0,1,2\ldots \right)$, we reset η such that V<η and S(·)<0. Since χ,ζ are continuous if η is not reset, there exists a small time interval $\left[t_{s}^{m},t_{s}^{m}+\iota \right)$ such that S(·)<0 holds where ι>0 is a small constant. This means that the switching signal σ(t) is right continuous, i.e., σ(t) will not change on $\left[t_{s}^{m},t_{s}^{m}+\iota \right)$. This will avoid the chattering phenomenon and guarantee that the switching times are strictly increasing.

The purpose of the algorithm is to let S(·)=V(·)−ζ(t)≤0 hold forever after *finite* switching times. Given that ζ(t) will become zero in finite time by (10), so will V(·) and χ.

Note that the finite switching times are possible because there exists a sufficiently large integer σ such that (8) holds when $\chi \in (-\bar{\chi },\bar{\chi })$. Then, by Comparison Principle and (10) with appropriate initial condition, we can show S(·)=V(·)−ζ(t)≤0 holds after finite switching times.

### 3.3. Stability Analysis

Based on the design in Section 3.1 and Section 3.2, we have the following result.

**Theorem** **1.** *Consider the nonlinear systems in (1). Then, the controller (4) with Algorithm 1 can make the state x converge to zero in finite time.*

**Proof.**  Define a time sequence $\left\{0=t_{s}^{0}<t_{s}^{1}<\ldots <t_{s}^{m}<\ldots \right\}$ with m∈{0,1,2,…}. $t_{s}^{m}$ denotes switching time, i.e.,

(11) $$t_{s}^{m+1}=\inf\left\{t|t\geq t_{s}^{m},S\left(\cdot \right)>0\right\}.$$

From Algorithm 1, we also know that this is the time instant when $\sigma ,\;\hat{\theta }$ update their values. Meanwhile, during time interval $\left[t_{s}^{m},t_{s}^{m+1}\right)$, no switching occurs and the supervisory function satisfies S(·)=V−ζ≤0. That is $\sigma ,\;\hat{\theta }$ remain to be constant. The proof is then divided into the following three claims. Figure 1 shows one possible variation of σ, V, ζ. □

**Claim a.** Given any finite integer *m*, on time interval $\left[t_{s}^{m},t_{s}^{m+1}\right)$, |x(t)|<χ and η, V are bounded.

**Proof.**  η is non-increasing on $\left[t_{s}^{m},t_{s}^{m+1}\right)$ because $\dot{\zeta }\left(t\right)=-a\zeta^{\gamma }\leq 0$ where we have used the fact that 0≤V≤η between two switching times. Moreover, from Algorithm 1 in Switching logic, at each switching time $t_{s}^{m}$, η will be increased by a finite value. Hence, we can conclude that ζ(t) is bounded on $\left[t_{s}^{m},t_{s}^{m+1}\right)$ for any finite *m*. Since 0≤V≤ζ on $\left[t_{s}^{m},t_{s}^{m+1}\right)$, *V* is bounded. Finally, according to the barrier Lyapunov function (2), we can conclude that $|\chi \left(t\right)|<\bar{\chi }$. □

**Claim b.** *The switching times are finite. Moreover, $|\chi \left(t\right)|<\bar{\chi }$ and ζ,V are bounded on [0,+∞).*

**Proof.**  This is proved by contradiction. If the claim is not true, then the switching times can be infinite. Note that by Claim a, $|\chi \left(t\right)|<\bar{\chi }$ on $\left[t_{s}^{m},t_{s}^{m+1}\right)$ with finite integer *m*. Meanwhile, σ and $\hat{\theta }$ are constants on $\left[t_{s}^{m},t_{s}^{m+1}\right)$ by construction. Therefore, Equations (6) and (7) in Section 3.1 hold. Meanwhile, there must exists a sufficiently large $m^{\prime }$ or σ such that (8) holds on $\left[t_{s}^{m^{\prime }},t_{s}^{m^{\prime }+1}\right)$. Since ζ is determined by (10) with $\zeta \left(t_{s}^{m^{\prime }}\right)>V\left(t_{s}^{m^{\prime }}\right)$, by Comparison Principle, we can see ζ will always be larger than *V* when $t\geq t_{s}^{m^{\prime }}$ without resetting ζ(t). This indicates that the switching times cannot be infinite, thereby contradicting the assumption. Since the switching times are finite, we can conclude $|\chi \left(t\right)|<\bar{\chi }$ and ζ, V are both bounded on [0,+∞). □

**Claim c.** *The state χ converges to zero in finite time.*

**Proof.**  Since the switching times are finite, then there exists a switching time $t_{s}^{m^{\prime }}$ such that 0≤V≤ζ always holds on $\left[t_{s}^{m^{\prime }},\infty \right)$. Solving (10), we have

(12) $$\begin{array}{cc} 0\leq & V\leq {\left[V^{1-\gamma }\left(t_{s}^{m^{\prime }}\right)-a\left(1-\gamma \right)\left(t-t_{s}^{m^{\prime }}\right)\right]}^{\frac{1}{1-\gamma }}. \end{array}$$

Noting $\zeta (t_{s}^{m^{\prime }})$ is bounded by Claim b, we can conclude *V* will converge to zero in finite time. □

**Remark** **1.** *Compared with the existing works [9,12], the main contributions of our proposed method lie on the following aspects: First, the proposed method can deal with fully unknown uncertainties f(χ) with unknown sign function b(χ). Second, the fast finite-time stability can be achieved by using the term $-K\chi^{\alpha }-L\chi^{\beta }$ in (4). The power terms β and α can improve the convergence speed.*

**Algorithm 1** Logic-based switching rule.*Initialization*
At t=0.
Set K,U, a,ε where ε is a small positive constant;Let σ(0)=1, $\bar{\chi }>\chi \left(0\right)$, ζ(0)=V(0)+ε;Implement control effort u(0) by (4).
*Switching logic*
**while**
t>0
Obtain the current states χ(t) and compute V, ζ, S by (2), (10) and (9);Check whether or not S(·)>0. If S(·)>0, go to 3); otherwise $\hat{\theta }$ is not updated, i.e., $\sigma \left(t\right)=\sigma \left(t^{-}\right)$, go to 5);If S(·)>0, let $\sigma \left(t\right)=\sigma \left(t^{-}\right)+1$ and compute $\hat{\theta }$ by (5);Update ζ(t)=V+ε to make S(·)<0;Use the updated parameter $\hat{\theta }$ to implement control effort *u* by (4).
**end**


## 4. Adaptive Sampled-Data Control for Nonlinear Systems

### 4.1. Problem Formulation

Consider the following nonlinear system

(13) $$\begin{array}{cc} {\dot{x}}_{i} & =x_{i+1}+f_{i}\left({\bar{x}}_{i}\right),\;i=1,2,\ldots ,n-1\\ {\dot{x}}_{n} & =u+f_{n}\left({\bar{x}}_{n}\right),\\ y & =x_{1} \end{array}$$

where ${\bar{x}}_{i}={\left(x_{1},x_{2},\ldots ,x_{i}\right)}^{T}\in R^{i}\left(i=1,2,\ldots ,n\right)$ are the states of each agent, $y_{i}$ is the output. $f_{i}\left({\bar{x}}_{i}\right)\left(i=1,\ldots ,n\right)$ are all unknown smooth nonlinear functions. It is assumed that $f_{i}\left({\bar{x}}_{i}\right)$ satisfies the linear growth condition such that $|f_{i}\left({\bar{x}}_{i}\right)\left|\leq \theta (\right|x_{1}\left|+\right|x_{2}\left|+\cdots +\right|x_{i}\left|\right)$ where θ is a positive constant representing the growth rate. The control objective is to design a sampled-data controller *u* such that all the states ${\bar{x}}_{n}$ will converge to zero exponentially.

### 4.2. Controller Design

Denote the sampling time instants as $\left\{t_{k}\right\}{}_{k=0,1,\ldots }$. Let $T_{k}=t_{k+1}-t_{k}>0$. Define an adaptive parameter $L_{k}\geq 1$ for time interval $\left[t_{k},t_{k+1}\right)$. $L_{k}$ keeps constant during time interval $\left[t_{k},t_{k+1}\right)$. The following controller design will be developed on $\left[t_{k},t_{k+1}\right)$.

**Step 1. Change of coordinates.**

Let

$$\begin{array}{cc} z_{i} & =\frac{x_{i}}{L_{k}^{i-1}},\;i=1,2,\ldots ,n,\\ v & =\frac{u}{L_{k}^{n}}. \end{array}$$

Then, we have

(14) $$\begin{array}{cc} {\dot{z}}_{i} & =L_{k}z_{i+1}+{\bar{f}}_{i}\left({\bar{z}}_{i}\right),\;i=1,2,\ldots ,n-1\\ {\dot{z}}_{n} & =L_{k}v+{\bar{f}}_{n}\left({\bar{z}}_{n}\right) \end{array}$$

where

$${\bar{f}}_{i}\left({\bar{z}}_{i}\right)=\frac{f_{i}\left({\bar{x}}_{i}\right)}{L_{k}^{i-1}}\left(i=1,2,\ldots ,n\right).$$

Based on the linear growth condition, we have

(15) $$\begin{array}{cc} |{\bar{f}}_{i}\left({\bar{z}}_{i}\right)| & \leq \frac{\theta (|x_{1}|+|x_{2}|+\cdots +|x_{i}|)}{L_{k}^{i-1}}\\ \; & =\theta \left(\frac{|z_{1}|}{L_{k}^{i-1}}+\frac{|z_{2}|}{L_{k}^{i-2}}+\cdots +\left|z_{i}\right|\right)\\ \; & \leq \theta (|z_{1}|+|z_{2}|+\cdots +|z_{i}|). \end{array}$$

**Step 2. Control law.**

The controller is designed as:

(16) $$\begin{array}{cc} v(t) & =-Kz(t_{k}), \end{array}$$

(17) $$\begin{array}{cc} u(t) & =-L_{k}^{n}Kz\left(t_{k}\right) \end{array}$$

where $K=\left[\kappa_{1}\;\kappa_{2}\;\ldots \;\kappa_{n}\right]\in R^{n}$ are coefficients of the Hurwitz polynomial $s^{n}+\kappa_{n}s^{n-1}+\cdots +\kappa_{2}s+\kappa_{1}$. $z\left(t\right)={\left[z_{1}\left(t\right)\;z_{2}\left(t\right)\;\ldots \;z_{n}\left(t\right)\right]}^{T}$.

Then, (14) becomes

(18) $$\begin{array}{c} \dot{z}\left(t\right)=L_{k}Az\left(t\right)+F\left(z\left(t\right)\right)-L_{k}BKz\left(t_{k}\right) \end{array}$$

where $F\left(z\left(t\right)\right)={\left[{\bar{f}}_{1}\left({\bar{z}}_{1}\right)\;{\bar{f}}_{2}\left({\bar{z}}_{2}\right)\;\ldots \;{\bar{f}}_{n}\left({\bar{z}}_{n}\right)\right]}^{T}$. $B={\left[0_{n-1}\;1\right]}^{T}$,

$$A=\left[\begin{array}{cc} 0_{n-1}^{T} & I_{n-1}\\ 0 & 0_{n-1} \end{array}\right].$$

Choose Lyapunov function

$$\begin{array}{cc} V(z(t)) & =z^{T}\left(t\right)Pz(t)\\ \; & =\left[x_{1}\;\frac{x_{2}}{L_{k}}\;\ldots \;\frac{x_{n}}{L_{k}^{n-1}}\right]P{\left[x_{1}\;\frac{x_{2}}{L_{k}}\;\ldots \;\frac{x_{n}}{L_{k}^{n-1}}\right]}^{T} \end{array}$$

where *P* is selected to satisfy ${\bar{A}}^{T}P+P\bar{A}=-I$ with $\bar{A}=A-BK$.

Differentiating it with time, we have

$$\begin{array}{cc} \dot{V} & =-L_{k}{\left||z|\right|}^{2}+2zPF\left(z\right)+2L_{k}zPBK\left(z\left(t\right)-z\left(t_{k}\right)\right). \end{array}$$

For 2zPF(z), using (15) we have

(19) $$\left||2zPF\left(z\right)|\right|\leq c_{1}{\left||z|\right|}^{2}$$

where $c_{1}>0$ is an unknown positive constant.

Then, we have

(20) $$\begin{array}{cc} \dot{V} & \leq -aV+\left(c_{2}-L_{k}\right){\left||z|\right|}^{2}+2L_{k}zPBK\left(z\left(t_{k}\right)-z\left(t\right)\right)\\ \; & =-aV+W(z\left(t\right),z\left(t_{k}\right),L_{k}) \end{array}$$

where a>0 is a positive constant, $c_{2}=a+c_{1}$ is an unknown positive constant.

(21) $$W\left(z\left(t\right),z\left(t_{k}\right),L_{k}\right)=\left(c_{2}-L_{k}\right){\left||z|\right|}^{2}+2L_{k}zPBK\left(z\left(t_{k}\right)-z\left(t\right)\right).$$

**Step 3. Logic-based switching law.**

Based on the above analysis, the switching law for $L_{k}$ and the sampling period $T_{k}=t_{k+1}-t_{k}$ are designed as follows:

(22) $$\begin{array}{cc} L_{k} & =\iota_{1}\sigma_{k}, \end{array}$$

(23) $$\begin{array}{cc} T_{k}L_{k}^{2} & \leq \iota_{2} \end{array}$$

where $\iota_{1},\iota_{2}$ are two positive constants. The signal $\sigma_{k}$ is given as follows.

For k=0, $\sigma_{0}$ is selected to be a positive constant such that $\iota_{1}\sigma_{0}\geq 1$.

For k≥1, $\sigma_{k}$ is governed by:

(24) $$\sigma_{k}=\left\{\begin{array}{cc} \sigma_{k-1}, & if\;S_{k}\leq 0;\\ \sigma_{k-1}+1, & if\;S_{k}>0 \end{array}\right.$$

where

(25) $$S_{k}=V(\tilde{z}\left(t_{k})\right)-e^{-T_{k-1}a}V\left(z\left(t_{k-1}\right)\right)$$

with

$$\tilde{z}(t_{k})={\left[x_{1}\left(t_{k}\right)\;\frac{x_{2}\left(t_{k}\right)}{L_{k-1}}\;\ldots \;\frac{x_{n}\left(t_{k}\right)}{L_{k-1}^{n-1}}\right]}^{T}.$$

The detail tuning law is given in Algorithm 2 in the table.
**Algorithm 2** Logic-based switching rule.*Initialization*
At $t_{0}=0$,
Choose $\sigma_{0}$ and $T_{0}$ such that $L_{0}=\iota_{1}\sigma_{0}\geq 1$, $T_{0}L_{0}^{2}\leq \iota_{2}$.Output the parameters $L_{0},T_{0}$.
*Switching logic*
At each sampling time $t_{k}>0\left(k=1,2,\ldots \right)$,
Compute $S_{k}$ by (25);If $S_{k}\leq 0$, $\sigma_{k}=\sigma_{k-1}$, $L_{k}=L_{k-1}$ and $T_{k}=T_{k-1}$ are not updated. Goto 5);If $S_{k}>0$, let $\sigma_{k}=\sigma_{k-1}+1$ and compute $L_{k}$ by (22).Check whether or not $T_{k}L_{k}^{2}\leq \iota_{2}$. If $T_{k}L_{k}^{2}>\iota_{2}$, choose a small sampling period $T_{k}$ to make $T_{k}L_{k}^{2}\leq \iota_{2}$, otherwise $T_{k}=T_{k-1}$ is not updated.Output the parameters $L_{k},T_{k}$.


### 4.3. Main Result and Stability Analysis

According to the controller designed in Section 3, we have the following result.

**Theorem** **2.** *Consider the nonlinear systems (13). Then, the controller law (16) and (17) with Algorithm 1 can guarantee the exponential stability, i.e., all the states will converge to zero exponentially.*

**Remark** **2.** *Compared with [13,21], the linear growth rate θ is unknown. This brings difficulties to selections of scaling gain $L_{k}$ and sample time $T_{k}$. Hence, we adaptively update $L_{k},T_{k}$ by Algorithm 1, which finally maintains the exponential stability of the closed loop system.*

The proof is divided into the following three claims. 

*Claim* 1. Given a finite *k*, then all the signals are bounded on $\left[t_{k},t_{k+1}\right)$.

**Proof.**  Based on (18), on time interval $\left[t_{k},t_{k+1}\right)$, we have

$$\begin{array}{cc} ||z\left(t\right)-z\left(t_{k}\right)\left|\right|\leq & L_{k}\varrho_{1}\int_{t_{k}}^{t}\left|\right|\left(z\left(t_{k}\right)-z\left(\tau \right)\right)\left|\right|d\tau \\ \; & +T_{k}L_{k}\varrho_{2}||z\left(t_{k}\right)\left|\right| \end{array}$$

where $\varrho_{1}=\left|\right|\bar{A}\left|\right|+\varrho_{3}+\left||BK|\right|$, $\varrho_{2}=\left|\right|\bar{A}\left|\right|+\varrho_{3}$, $\varrho_{3}$ is a positive constant such that $\left||F\left(z\right)|\right|\leq \varrho_{3}\left||z|\right|$.

By Gronwall’s inequality, we have

(26) $$\begin{array}{cc} ||z\left(t\right)-z\left(t_{k}\right)\left|\right| & \leq \left(T_{k}L_{k}\varrho_{2}+T_{k}^{2}L_{k}^{2}\varrho_{1}\varrho_{2}e^{T_{k}L_{k}\varrho_{2}}\right)||z\left(t_{k}\right)\left|\right|\\ \; & =\xi \left(L_{k},T_{k}\right)||z\left(t_{k}\right)\left|\right|. \end{array}$$

where

(27) $$\xi \left(L_{k},T_{k}\right)=T_{k}L_{k}\varrho_{2}+T_{k}^{2}L_{k}^{2}\varrho_{1}\varrho_{2}e^{T_{k}L_{k}\varrho_{2}}.$$

From (22) and (23), it can be seen that

(28) $$\begin{array}{c} \xi \left(L_{k},T_{k}\right)\leq \frac{\iota_{2}\varrho_{2}}{L_{k}}+\frac{\iota_{2}^{2}\varrho_{1}\varrho_{2}}{L_{k}^{2}}e^{\frac{\iota_{2}\varrho_{2}}{L_{k}}}\leq \varrho_{4}-1 \end{array}$$

where $\varrho_{4}>0$ is a positive constant.

Hence, from (26)

(29) $$\begin{array}{cc} \left||z(t)|\right| & \leq \varrho_{4}||z\left(t_{k}\right)\left|\right|,\;\forall t\in \left[t_{k},t_{k+1}\right). \end{array}$$

Also note that at time instant $t_{k}$ and $t_{k}^{-}\left(k=1,2,\ldots \right)$, we have

$$\begin{array}{cc} z(t_{k}) & =M_{k}x\left(t_{k}\right),\\ z(t_{k}^{-}) & =M_{k-1}x\left(t_{k}^{-}\right) \end{array}$$

where $M_{k}=\mathrm{diag}\left[1,\frac{1}{L_{k}},\ldots ,\frac{1}{L_{k}^{n-1}}\right].$

Due to x(t) is continuous, this implies that there is a finite positive constant $\Delta_{k}$ such that

(30) $$||z\left(t_{k}\right)\left|\right|\leq ||z\left(t_{k}^{-}\right)\left|\right|+\Delta_{k}.$$

Then, using (29) and (30) repeatedly, we obtain:

$$\begin{array}{cc} \left||z(t)|\right| & \leq \varrho_{4}||z\left(t_{k}^{-}\right)\left|\right|+\varrho_{4}\Delta_{k}\\ \; & \leq \varrho_{4}^{2}||z\left(t_{k-1}\right)\left|\right|+\varrho_{4}\Delta_{k}\\ \; & \leq \cdots \\ \; & \leq \varrho_{4}^{k+1}||z\left(t_{0}\right)\left|\right|+\sum_{i=1}^{k}\varrho_{4}^{i}\Delta_{k-i+1} \end{array}$$

Note that $z(t_{0})$ is bounded. Hence, z(t) will be bounded as long as *k* is finite. The proof is completed. □

*Claim* 2. The number of switching times is finite.

**Proof.**  By contradiction, if this is not true, then the number of switching times is infinity. According to (22) and (24), we know $L_{k}$ can any large number. Using (28), we know there exists a sufficiently large $k^{\prime }$ such that

$$\xi \left(L_{k},T_{k}\right)\leq 1-\varrho_{5}$$

for $k\geq k^{\prime }$ where $0<\varrho_{5}<1$ is a positive constant.

Then, (26) becomes

$$\begin{array}{cc} ||z\left(t_{k}\right)-z\left(t\right)\left|\right| & \leq \xi \left(L_{k},T_{k}\right)||z\left(t_{k}\right)\left|\right|\\ \; & \leq \xi ||z\left(t\right)-z\left(t_{k}\right)\left|\right|+\xi ||z\left(t\right)\left|\right|. \end{array}$$

Then, we have

$$\begin{array}{cc} ||z\left(t_{k}\right)-z\left(t\right)\left|\right| & \leq \bar{\xi }\left(L_{k},T_{k}\right)||z\left(t\right)\left|\right|\leq \frac{\xi }{\varrho_{5}}||z\left(t\right)\left|\right| \end{array}$$

where $\bar{\xi }=\xi /\left(1-\xi \right).$

Also note that using (27), we have

(31) $$\begin{array}{cc} L_{k}||z\left(t_{k}\right)-z\left(t\right)\left|\right|\leq & \frac{L_{k}\xi }{\varrho_{5}}\left||z\left(t\right)|\right|\\ \leq & \left(\frac{\iota_{2}\varrho_{2}}{\varrho_{5}}+\frac{\iota_{2}^{2}\varrho_{1}\varrho_{2}}{L_{k}}e^{\frac{\iota_{2}\varrho_{2}}{L_{k}}}\right)\left||z\left(t\right)|\right|\\ \leq & \varrho_{6}\left||z\left(t\right)|\right| \end{array}$$

where $\varrho_{6}$ is a positive constant. Hence, substituting (31) into W(·) in (21), we have

$$\begin{array}{c} W\left(\cdot \right)\leq (c_{2}+2\varrho_{6}\left||PBK|\right|-L_{k}{)||z||}^{2}. \end{array}$$

It follows that there exists a $k^{\prime \prime }\geq k^{\prime }$ such that $c_{1}+2C||PBK||-L_{k}\leq 0$ for $k\geq k^{\prime \prime }$. From (20), we know

$$\begin{array}{cc} \dot{V} & \leq -aV \end{array}$$

for $k\geq k^{\prime \prime }$. In other words,

$$\begin{array}{cc} V & \left(z\left(t\right)\right)\leq V\left(z\left(t_{k}\right)\right)e^{-a(t-t_{k})} \end{array}$$

for $t\in [t_{k},t_{k+1})$ with $k\geq k^{\prime \prime }$.

This means $S_{k}\leq 0$ for $k\geq k^{\prime \prime }$. Hence, the switch will stop for $t\in (t_{k^{\prime \prime }},+\infty )$. This contradicts the fact that there is an infinite number of switchings. The proof is completed. □

*Claim* 3. Exponential stability is achieved.

**Proof.**  Suppose at time instant $t_{k^{\prime \prime }}$, the switch stops.

Then, during time interval $\left[0,t_{k^{\prime \prime }}\right)$, according to Claim 1, we have ||z(t)|| is bounded on $\left[0,t_{k^{\prime \prime }}\right)$.

During time interval $\left[t_{k^{\prime \prime }},+\infty \right)$, since no switching happens, we have $L_{k}=L_{k^{\prime \prime }}$, $T_{k}=T_{k^{\prime \prime }}$, $S_{k}\leq 0$ for $k\geq k^{\prime \prime }$. Hence, for any $t\in [t_{k},t_{k+1})$ with $k\geq k^{\prime \prime }$, by (25), we have

$$\begin{array}{cc} V(z\left(t_{k})\right) & \leq e^{-T_{k^{\prime \prime }}a}V\left(z\left(t_{k-1}\right)\right).\\ \; & \leq \cdots \leq e^{-\left(k-k^{\prime \prime }\right)T_{k^{\prime \prime }}a}V\left(z\left(t_{k^{\prime \prime }}\right)\right) \end{array}$$

This means that as t→+∞, k→+∞, we have $V(z\left(t_{k})\right)\to$0. Hence, $\left||z(t_{k})|\right|$ converges to zero exponentially. By (29), we know ||z(t)|| will converge to zero exponentially. The proof is completed. □

**Remark** **3.** *
In contrast with the existing works [12,13], the presented method can handle the unknown linear growth rate θ in (13). Moreover, the sampling period $T_{k}$ can be adjusted adaptively. This can reduce the conservatism and save the time of tuning the sampling period.
*

## 5. An Illustrative Example

**Figure 2:**
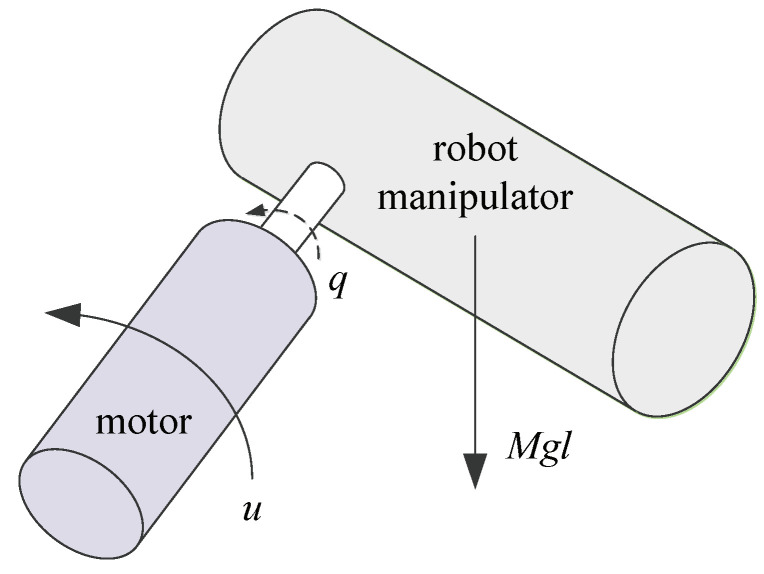
Robot manipulator.

**Example** **1.** *Consider a robot manipulator described by (13) with n=2 in Figure 2. The nonlinear functions are given by $f_{1}\left(x_{1}\right)=0.1\sin\left(x_{1}\right)$, $f_{2}\left({\bar{x}}_{2}\right)=-0.5MgL\sin\left(x_{1}\right)/J+0.05\sin\left(x_{1}\right)e^{-x_{2}}+0.1\sin\left(x_{2}\right)$. M=1kg, $g=9.8\;m/s^{2}$, L=1m, $J=1\;\mathrm{kg}\cdot m^{2}$.*

We will adopt the controller in (4). The controller parameters are selected as K=L=U=1, α=39/49, $\bar{\chi }=5$, a=0.2.

**Figure 3:**
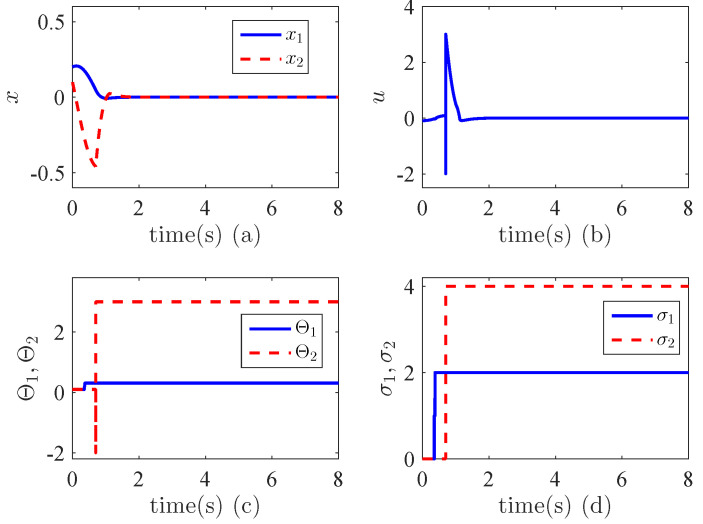
Finite time stabilization in Example 1. (**a**) State trajectories; (**b**) Control effort; (**c**) Variations of adaptive parameters; (**d**) Switching signals.

**Figure 4:**
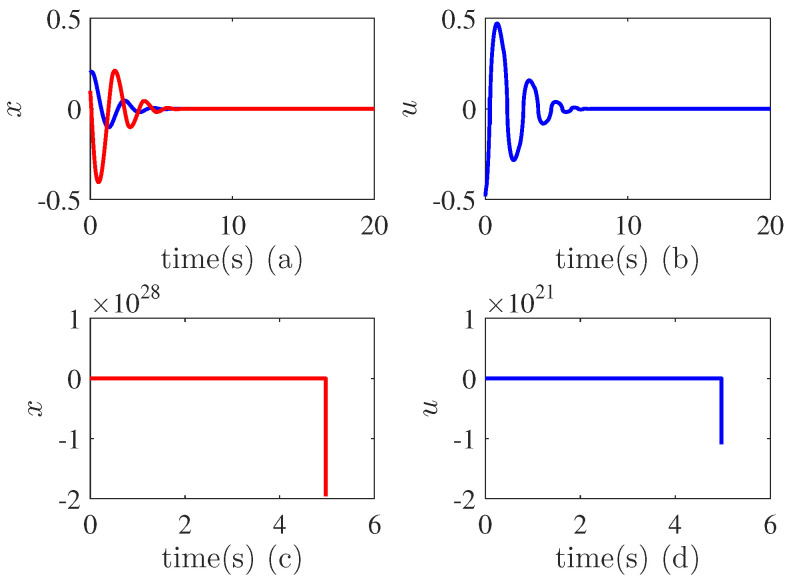
Comparisons in Example 1. (**a**) State trajectories when the control gain is negative. Blue line denotes state $x_{1}$, red line denotes $x_{2}$; (**b**) Control effort when the control gain is negative; (**c**) State trajectories when the control gain is positive. Blue line denotes state $x_{1}$, red line denotes $x_{2}$; (**d**) Control effort when the control gain is positive.

From Figure 3a, it can be seen that the states converge to zero in a very short time, i.e., the output $|x_{1}|$ is around $3\times 10^{-14}$ after 6 s. This implies that the finite time stability is achieved. Figure 3c shows the variations of the parameters $\theta_{1},\theta_{2}$. We can see that they are tuned adaptively to due to the uncertainties and unknown control directions. Figure 3d illustrates the variations of the switching signal $\sigma_{1}\left(t\right)$, $\sigma_{2}\left(t\right)$, which indicates that they are bounded. All these verify the validity the proposed method. We have compared our method with the non-adaptive finite-time controller. Figure 4a,b shows the results when the control gain is negative while Figure 4c,d demonstrates the performance when the control direction is positive. We can see that in both cases, the control performance is not better than the proposed adaptive controller. In fact, when the control direction does not match the control gain, the system becomes unstable.

**Figure 5:**
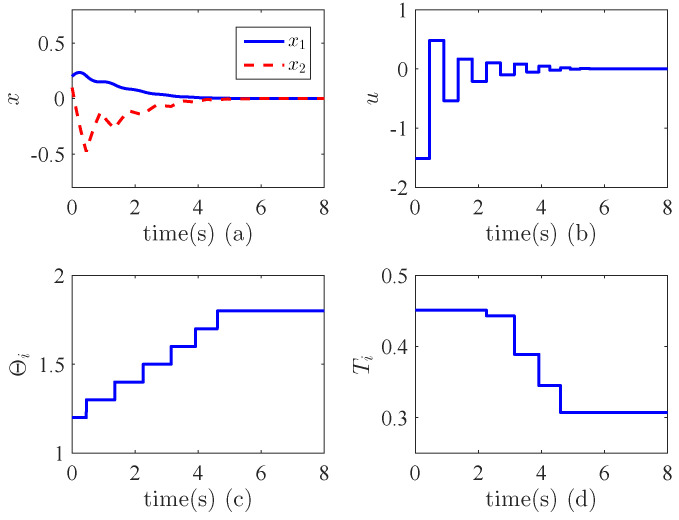
Adaptive sampled data stabilization. (**a**) State trajectories; (**b**) Control effort; (**c**) Variations of adaptive parameters; (**d**) Variations of sampling time period.

**Figure 6:**
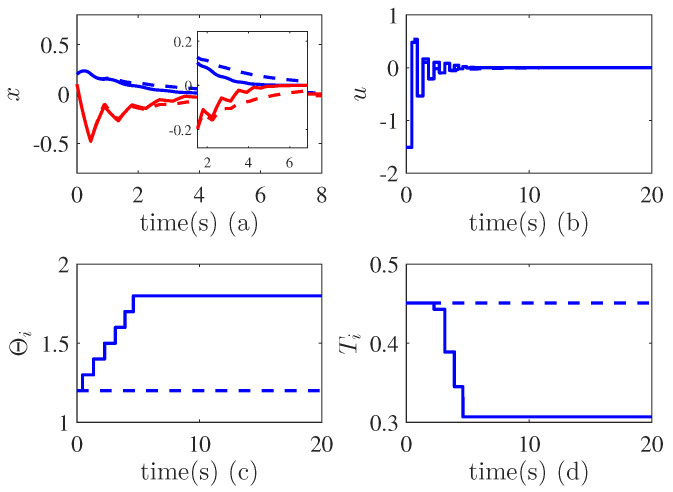
Comparison in Example 1. (**a**) State trajectories. Blue line denotes state $x_{1}$, red line denotes $x_{2}$; (**b**) Control effort; (**c**) Variations of adaptive parameters; (**d**) Variations of sampling time period. Dashed line is non-adaptive controller, solid line is the proposed controller.

**Example** **2.** Consider a mechatronic system described by (13) with n=2. The nonlinear functions are given by $f_{1}\left(x_{1}\right)=\sin\left(x_{1}\right)$, $f_{2}\left({\bar{x}}_{2}\right)=-2x_{2}+\sin\left(x_{2}\right)+\sin\left(x_{1}\right)\ln\left(1+x_{2}^{2}\right)$. The controller parameters are selected as K=[43], $L_{0}=1.2$, $T_{0}=0.45$ s, ι=0.1, a=0.1, $c^{*}=1$. Figure 5a shows the the states converge to zero at around 6 s, which indicates that the the system is stable. Figure 5c,d illustrate the variations of the control parameter $L_{k}$ and sampling period $T_{k}$. We can see that $L_{k}$ and $T_{k}$ become large and small enough separately in order to guarantee the stability of the closed loop system. This verifies the effectiveness of the proposed controller. We have compared our method with the non-adaptive sampled-data controller. As shown in Figure 6, the proposed method has a faster convergence speed than the non-adaptive controller.

## 6. Conclusions

In this paper, we have considered two different classes of stabilization problem. In the firFigurest case, a logic-based switching adaptive control method is proposed, which can handle both unknown nonlinearities and control directions. In the second case, the proposed method can handle uncertain linear growth rate. The control parameters and the sampling time can be adjusted adaptively to render the exponential stability of the closed loop system. Applications in robot manipulators are conducted to verify the proposed results. Future works include considering applications in more complex robots.

## References

[1] Karafyllis I., Krstic M. (2018). Adaptive Certainty-Equivalence Control with Regulation-Triggered Finite-Time Least-Squares Identification. IEEE Trans. Autom. Control.

[2] Tao G. (2014). Multivariable adaptive control: A survery. Automatica.

[3] Qiao W., Tang X., Zheng S., Xie Y., Song B. (2016). Adaptive two-degree-of-freedom PI for speed control of permanent magnet synchronous motor based on fractional order GPC. ISA Trans..

[4] Wang J.-W., Liu Y.-Q., Sun C.-Y. (2018). Adaptive Neural Boundary Control Design for Nonlinear Flexible Distributed Parameter Systems. IEEE Trans. Control Syst. Technol..

[5] Smyshlyaev A., Krstic M. (2010). Adaptive Control of Parabolic PDEs.

[6] Li Y., Liu L., Feng G. (2018). Robust adaptive output feedback control to a class of non-triangular stochastic nonlinear systems. Automatica.

[7] Yu J., Shi P., Zhao L. (2018). Finite-time command filtered backstepping control for a class of nonlinear systems. Automatica.

[8] Zheng S., Li W. (2018). Fuzzy Finite Time Control for Switched Systems via Adding a Barrier Power Integrator. IEEE Trans. Cybern..

[9] Chen W., Wen C., Wu J. (2018). Global Exponential/Finite-Time Stability of Nonlinear Adaptive Switching Systems with Applications in Controlling Systems with Unknown Control Direction. IEEE Trans. Autom. Control.

[10] Hespanha J.P., Liberzon D., Morse A. (2003). Overcoming the limitations of adaptive control by means of logic-based switching. Syst. Control Lett..

[11] Kersting S., Buss M. (2017). How to Systematically Distribute Candidate Models and Robust Controllers in Multiple-Model Adaptive Control: A Coverage Control Approach. IEEE Trans. Autom. Control.

[12] Wu J., Chen W., Li J. (2016). Global finite-time adaptive stabilization for nonlinear systems with multiple unknown control directions. Automatica.

[13] Qian C., Du H. (2012). Global output feedback stabilization of a class of nonlinear systems via linear sampled-data control. IEEE Trans. Autom. Control.

[14] Huang S., Xiang Z. (2016). Finite-time stabilization of switched stochastic nonlinear systems with mixed odd and even powers. Automatica.

[15] Li L., Wang D. (2022). On Finite/Fixed-Time Stability Theorems of Discontinuous Differential Equations. Mathematics.

[16] Yue Y., Geng Y., Wang W. (2022). Continuous Nonsingular Fast Terminal Sliding Mode Control for Speed Tracking of PMSM Based on Finite Time Disturbance Observer. Processes.

[17] Kong L., He W., Yang W., Li Q., Kaynak O. (2020). Fuzzy Approximation-Based Finite-Time Control for a Robot with Actuator Saturation under Time-Varying Constraints of Work Space. IEEE Trans. Cybern..

[18] Lv M., Yu W., Cao J., Baldi S. (2020). Consensus in High-Power Multiagent Systems with Mixed Unknown Control Directions via Hybrid Nussbaum-Based Control. IEEE Trans. Cybern..

[19] Liang B., Zheng S., Ahn C.K., Liu F. (2020). Adaptive Fuzzy Control for Fractional-Order Interconnected Systems with Unknown Control Directions. IEEE Trans. Fuzzy Syst..

[20] Zhang J., Dai X. (2022). Adaptive Fuzzy Control for Flexible Robotic Manipulator with a Fixed Sampled Period. Electronics.

[21] Lin W., Wei W. (2018). Semi-global asymptotic stabilization of lower triangular systems by digital output feedback. IEEE Trans. Autom. Control.

